# The Broadleaf Weeds Control Efficiency of Drip Irrigation Herbicides in Cotton Fields and the Cotton Safety Assessment

**DOI:** 10.3390/plants14111589

**Published:** 2025-05-23

**Authors:** Ruitong Yang, Jiayi Zhang, Sen Wang, Gulfam Yousaf, Hao Tan, Lixing Yang, Muhammad Zeeshan, Cailan Wu, Desong Yang

**Affiliations:** 1 Key Laboratory of Oasis Agricultural Disease and Pest Management and Plant Protection Resource Utilization in Xinjiang, School of Agriculture, Shihezi University, Shihezi 832000, China; yangruitong@stu.shzu.edu.cn (R.Y.); 20221012195@stu.shzu.edu.cn (J.Z.); wangsen@stu.shzu.edu.cn (S.W.); gulfamyousaf0052@gmail.com (G.Y.); maharzeeshan218@gmail.com (M.Z.); 2 College of Water Conservancy & Architectural, Shihezi University, Shihezi 832000, China; 20211013167@stu.shzu.edu.cn (H.T.); 20241006164@stu.shzu.edu.cn (L.Y.)

**Keywords:** drip irrigation, herbicide residues, broadleaf weeds, cotton safety

## Abstract

The aim of this study is to precisely elucidate the control efficacy of drip irrigation herbicide application against broadleaf weeds and comprehensively assess its safety to cotton. Broadleaf weeds were managed through the application of herbicide in the cotton field. The herbicide was dispensed from a fertilizer tank in tandem with water droplets. A field investigation was conducted via a fixed-point investigation method to assess the herbicide residue levels and the safety of the cotton crop from 2022 to 2023. When 100.8 g a.i./hm^2^ of 48% Flumioxazin SC was applied via drip irrigation, it had no adverse effect on cotton safety at the mature stage. During the fruit-setting stage, it exhibited a significant weeding effect on annual broadleaf weeds such as *Solanum nigrum* L. and *Chenopodium album* L. Analysis revealed no pesticide residues in cotton and cottonseeds. Soil pesticide residues were found to be at a low level. The cotton yield reached 5618.1 kg/hm^2^, and the cotton quality met the national standard requirements. For the control of broadleaf weeds in cotton fields, the application of 100.8 g a.i./hm^2^ of 48% Flumioxazin SC via drip irrigation can effectively control broadleaf weeds. This method can suppress annual broadleaf weeds, with *S. nigrum* and *C. album* being the dominant weed communities, without compromising the safety and quality of cotton. Although drip irrigation technology offers advantages such as time savings and reduced labor demands, it is essential to adopt appropriate weed control techniques tailored to the specific conditions of different cotton fields.

## 1. Introduction

Xinjiang serves as a crucial cotton production base in China. The cotton produced here is renowned for its high yield and excellent quality. Nevertheless, weed infestation has long been one of the significant bottlenecks that impede the further enhancement of cotton yield and quality. The competition between weeds and cotton crops for water, nutrients, and light energy severely restricts the growth and productivity of cotton [1,2]. Among the various types of weeds, broadleaf weeds, particularly *Solanum nigrum* L., *Chenopodium album* L., and *Convolvulus arvensis* L., inflict substantial economic losses in cotton fields [3]. The root systems of these broadleaf weeds are well developed, and their ability to absorb soil moisture and nutrients far exceeds that of cotton plants. Enjoying significant growth advantages, they often outgrow cotton plants in height, seriously jeopardizing cotton’s nutrient absorption and photosynthesis processes and thus threatening the overall performance of the crop. It is evident that these weeds can be detrimental, both by competition and other threats, such as allelopathy and increased pressure of some pests and disease-causing organisms. Overwintering weeds in cotton or other rotational crops cause rapidly increasing harm during the following year [4]. Regarding cotton cultivation, several countries have embraced herbicide-tolerant transgenic crops. However, the over-reliance on herbicides for weed control in both transgenic and conventional farming systems has accelerated the evolution of herbicide-resistant weeds [5]. The coexistence of a large number of weeds and cotton greatly complicates the harvesting process for cotton pickers or machinery. This not only reduces the efficiency of mechanical harvesting but also increases the impurity content in seed cotton [6]. In conclusion, the presence of weeds in cotton fields has a profound impact on the yield and quality of cotton. In Xinjiang’s cotton fields, the average annual weed coverage is 56%, resulting in an annual yield reduction of approximately 13–15% [7,8].

Cotton exhibits high sensitivity to weeds during its early growth stage [9]. Consequently, chemical weed control is mainly implemented for soil treatment prior to cotton sowing. However, this treatment can only suppress weeds for approximately 45–60 days and is ineffective against weeds that germinate in the later stage. The prolonged presence of weeds in the cotton field beyond the critical period typically affects the height of cotton plants and results in a significant decline in cotton yield [10,11]. In fact, improper weed management in the cotton field can lead to yield losses of up to 90% [5]. Currently, there is no selective stem-spray treatment herbicide available for controlling broadleaf weeds in cotton fields. Some resistant weeds can be treated using the directional spray method. Nevertheless, this approach is time-consuming, labor-intensive, and incurs high economic costs. Additionally, the application of herbicides through foliar spraying in cotton fields can also cause phytotoxicity. Therefore, exploring effective methods for controlling broadleaf weeds during the middle and later stages of cotton growth is a crucial technology for enhancing the efficient production of cotton in the absence of suitable post-emergence herbicides.

Drip irrigation pesticide application technology represents a novel agricultural technique that integrates irrigation with the delivery of pesticides [12]. Its principle involves leveraging the pressure within the irrigation system to enable the rapid, uniform, and precise transportation of pesticides along with the irrigation water to the crop rhizosphere, thereby achieving the objective of controlling diseases, pests, and weeds [13,14]. Xinjiang is the largest cotton-producing region in China. Drip irrigation and high-density cultivation are conducive to the development of integrated cotton water, fertilizer, and pesticide technologies. This not only saves human and material resources but also enhances cotton yields [15]. Incorporating pesticides into the drip irrigation system of water–fertilizer integration can achieve the goal of delivering irrigation water, fertilizers, and pesticides directly to crops, thereby improving the utilization efficiency of pesticides and fertilizers. In this study, three types of herbicides, which were previously applied through soil spraying before cotton sowing, were introduced via irrigation water. This study aimed to evaluate the weed control effectiveness, assess the safety of cotton, and measure the yield, quality, and pesticide residues in cotton after application. The results offer both theoretical and practical implications for improving weed management in cotton fields.

## 2. Materials and Methods

### 2.1. Materials

Materials included the following: 48% Flumioxazin SC (Jiangsu Yongan Chemical Co., Ltd., Huaian, China), 50% Prometryn SC (Qiaochang Modern Agriculture Co., Ltd., Binzhou, China), and 42% Fluridone SC (Max (Rudong) Chemical Co., Ltd., Nantong, China); Flumioxazin, Prometryn, and Fluridone (Sourced by Tianjin Ministry of Agriculture, Tianjin, China); a small fertilization tank (50 L) and chromatographically pure methanol, acetonitrile, and formic acid (CNW Technologies GmbH Company, Dusseldorf, Germany); QuEChERS extraction salt package, QuEChERS dispersive SPE reagent, and ceramic proton (Agilent Technologies, Santa Clara, CA, USA).

### 2.2. Instruments

Ultra-high-performance liquid chromatography triple quadrupole mass spectrometer (Agilent Technologies, Santa Clara, CA, USA); Waters Acquity UPLC/Xevo-TQS (Waters, Milford, MA, USA); SIGMA3-30K centrifuge (Sigma, St. Louis, MO, USA); Ulupure water device (Sichuan Youpu Co., Ltd., Chengdu, China); BSA224S ten-thousandth balance (Sartorius, Goettingen, Germany); IQ7000 Ultrapure Water Meter (Millipore, Billerica, MA, USA); Jingda freezing water bath oscillator SHA-2A (Jintan Jingda Instrument Manufacturing Co., Ltd., Changzhou, China); G560E vortex oscillator (Scientific Industries, Bohemia, NY, USA).

### 2.3. Method

A field experiment was conducted from 2023 to 2024 at the Shihezi university experimental field (44°19′27″, 85°58′52″) to evaluate weed control with herbicides and safety to cotton. The soil type was loamy (alkalin) with a pH of 8.2 to 8.3, 23.06 g/kg organic matter, 19.20 mg/kg of phosphorus, and 311.22 mg/kg of potassium. The treatments were arranged in a completely randomized block design with the main experimental plots consisting of cotton cultivar Huiyuan 720 with Fluridone, Prometryn, and two concentrations of Flumioxazin applied on 25 July 2022 and 2023, 2 August 2022, and 2023, respectively. All 12 treatments were replicated three times. A set of untreated plants was included as the control.

In the drip irrigation method, a small fertigation tank was employed to deliver herbicides. The herbicides were thoroughly dissolved in the fertigation tank after undergoing two rounds of dilution. The pressure difference of field irrigation is exerted on the soil via three drip irrigation strips (capillary tubes) in each plot. During the drip irrigation process, clear water is dripped initially to achieve the objectives of flushing the drip irrigation system and moistening the soil. The chemical treatment is dripped 1.5 h prior to the end of drip irrigation, followed by 0.5 h of dripping clear water. This ensures that the chemicals in the drip irrigation belt are fully applied to the field soil along with the flowing water. Each drip irrigation belt in the drip irrigation experiment represents a replication, and the treated plot size at each location was a 2.05 × 600 m square, where 2.05 m was the film width (Figure S1). Each drug test treatment was repeated 3 times. In the weed control experiment, we conducted a weed base survey on 24 July 2022 and 2023, respectively. Fertilizer, irrigation, and insecticides were applied by the grower based on standard practices (Table S1). The specific test sites, test agents, pre-sowing weeding details, field test conditions, and information on weeds are presented in Table 1 and Table 2.

### 2.4. Experimental Investigation and Detection

#### 2.4.1. Weed Control Effect Investigation

The visual observation method was employed for the investigation. Each survey quadrat was 0.5 × 0.5 m. Four surveys were carried out in each plot, and each treatment was marked at a fixed point. The basic number of weeds was surveyed before the application of the herbicide. After the herbicide was applied, the weeds at the marked fixed points were investigated. In accordance with the criteria for pesticidal field efficacy tests (2) GB/T 17980.128-2004, the calculation method for the control effect is as follows:
Control efficiency on weeds (%) = (1 − number of weeds after spraying/number of weeds before spraying) × 100%Cotton number/hm^2^ = cotton number × 667/plot areaTheoretical yield (kg/hm^2^) = cotton number/hm^2^ × effective boll number per plant × single boll weight × 10^−3^

All data analyses were performed using IBM SPSS Statistics 22 software. Variance analysis was performed on all the data obtained from the test, and Fisher’s Protected LSD (*p* = 0.01, *p* = 0.05) was used for mean separation.

#### 2.4.2. Safety Investigation of Cotton

Grading method of cotton phytotoxicity:

Grade 1: normal growth of cotton without any symptoms of damage;

Grade 2: cotton mild phytotoxicity, phytotoxicity less than 10%;

Grade 3: moderate pesticide damage to cotton, which can be restored later without affecting yield;

Grade 4: cotton injury is serious, difficult to recover, and results in yield reduction;

Grade 5: cotton injury is serious, cannot be restored, and results in a significant reduction or no production.

#### 2.4.3. Detection of Pesticide Residues in Samples

##### Sample Extraction

Weigh 10 g of homogenized leaf, soil, or cottonseed samples into a 50 mL centrifuge tube. Add 20 mL of acetonitrile extract; then, vortex and oscillate the mixture for 5 min to ensure thorough mixing of the sample and the extraction solvent. Subsequently, add an extraction salt bag and a ceramic rotor, vortex for 1 min, and centrifuge at 4000 r/min for 5 min for purification purposes.

##### Purification of the Extract

After centrifuging for 5 min, transfer 10 mL of the supernatant into a 50 mL centrifuge tube containing a dispersed SPE reagent (purification kit). Vortex for 1 min and then centrifuge at 5000 r/min for 5 min. Collect the supernatant and filter it through a 0.22 µm organic membrane for subsequent HPLC-MS/MS analysis.

#### 2.4.4. Detection Conditions

##### Chromatographic Conditions

For Flumioxazin, Prometryn, and Fluridone, a water BEH C18 chromatographic column (50 mm × 2.1 mm, 1.8 µm) was employed. Mobile phase A consisted of a 0.1% formic acid aqueous solution, while mobile phase B was acetonitrile. Isocratic elution was carried out with a composition of 40% mobile phase A and 60% mobile phase B. The flow rate was set at 0.4 mL/min. The column temperature was maintained at room temperature, and the injection volume was 5 µL.

##### Mass Spectrometry Method

An electrospray ion source (ESI) was utilized, with nitrogen serving as both the collision gas and desolvation gas. The positive ion scanning multiple reaction monitoring (MRM) mode was adopted, and the residence time of the target compound was 20 ms. The atomizing gas was 99.95% nitrogen, and the collision gas was 99.999% nitrogen. The capillary voltage was set at 3.5 kV, the ion source temperature was 150 °C, the desolvation gas temperature was 500 °C, and the desolvation gas flow rate was 1000 L/min.

#### 2.4.5. Accuracy, Precision, and Sensitivity

The accuracy of the residue test was quantified by the recovery rate. Standard solutions of Flumioxazin, Prometryn, and Fluridone were spiked into the blank matrix at two distinct concentration levels, namely 10 µg/mL and 100 µg/mL. Each concentration level was replicated seven times (*n* = 7). The extraction and purification procedures were conducted in accordance with the method described in Section 2.4.3. Subsequently, the average recovery rate was computed according to the conditions specified in Section 2.4.4. The precision was evaluated by calculating the relative standard deviation (RSD, *n* = 7).

## 3. Results and Analysis

### 3.1. Drip Irrigation Herbicides Selectivity to Cotton

As presented in Table 3, within seven days following the drip irrigation application of the three tested herbicides, no phytotoxicity symptoms were detected in the cotton plants. However, ten days post-treatment, the application of 48% Flumioxazin SC at a dosage of 50.4 g a.i./hm^2^ induced mild secondary phytotoxicity in cotton. Specifically, approximately 10% of the upper leaves exhibited a slight reddening, while the lower leaves remained unaffected. When the dosage was increased to 100.8 g a.i./hm^2^, 48% Flumioxazin SC caused moderate phytotoxicity in some cotton plants, with 20% of the upper leaves and their veins turning red. Regarding 42% Fluridone SC, at a dosage of 252 g a.i./hm^2^, it caused moderate phytotoxicity to cotton. Approximately 20% of the upper leaves turned red and curled downward, while the lower leaves maintained their normal state. For 50% Prometryn SC, when applied at 900 g a.i./hm^2^, it led to more prominent phytotoxicity in cotton, with 40% of the upper leaves showing a slight reddening. The degree of pesticide damage to cotton was evaluated ten days after the drip irrigation application of the herbicides. The results demonstrated that among the three herbicides, 48% Flumioxazin SC caused the least degree of pesticide damage to cotton plants. Additionally, it was evident that higher concentrations of the herbicides were more likely to cause greater pesticide damage.

### 3.2. Symptoms and Control Effect of Weeds in Cotton Field After Drip Irrigation

As presented in Table 4, after 7 days of drip irrigation application, the control efficacy of 48% Flumioxazin SC exceeded 65%. Regarding *S. nigrum* with a plant height of 1–1.2 m, the application of 48% Flumioxazin SC led to significant damage symptoms. These included yellowing and wilting of the stem, and the upper leaves completely dried up, leaving only the basal green leaves. In contrast, for *S. nigrum* with approximately 10 leaves, the application of 48% Flumioxazin SC caused the entire plant to wilt and undergo necrosis.

**Figure 1:**
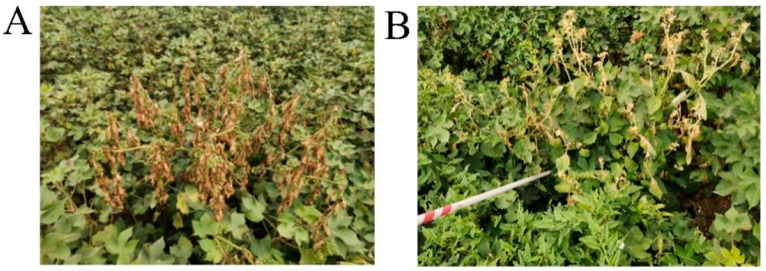
(**A**) *S. nigrum* after drip irrigation application of 48% Flumioxazin SC for 7 days. (**B**) *S. nigrum* after drip irrigation application of 42% Fluridone SC for 7 days.

For *C. album*, the control effect was relatively less pronounced. The stem turned red and showed slight wilting, yet the plant did not die. Moreover, 48% Flumioxazin SC had no control effect on *C. arvensis*, as depicted in Figure 1.

Further investigation indicated that the control efficacy of 50% Prometryn SC at a dosage of 900 g a.i./hm^2^ on weeds was 61.11%. For *S. nigrum* with approximately 10 leaves, the plant began to wilt and dry out, with clear signs of necrosis, while it had no impact on *C. arvensis*.

On the other hand, 42% Fluridone SC at a dosage of 252 g a.i./hm^2^ exhibited no control effect on weeds after 7 days. The heart leaves of *S. nigrum* and *C. album* turned yellowish-white, but no plant mortality was recorded. Additionally, the upper leaves of *S. nigrum* showed signs of desiccation, as shown in Figure 1.

After 7 days of application, a comparison of the control effects of the three herbicides on cotton-field weeds was conducted. It was found that among the three tested herbicides, 48% Flumioxazin SC demonstrated the most effective control effect on cotton-field weeds.

After 16 days of field investigation, the control efficacy of 48% Flumioxazin SC at a dosage of 50.4 g a.i./hm^2^ and 50% Prometryn SC exceeded 63%. Specifically, the control efficacy of 48% Flumioxazin SC at 100.8 g a.i./hm^2^ reached as high as 70%. The results indicated that this treatment inflicted significant damage on *S. nigrum* and *C. album*. Both of these weed species dried out and exhibited necrosis.

Notably, no control effect was detected on *C. arvensis*, despite the observation of a small amount of newly emerged *S. nigrum* and *C. arvensis* on the ground surface.

Regarding 42% Fluridone SC, its control efficacy reached 69% after 16 days. Within 7 days, the weeds showed chlorosis and bleaching, and subsequently, the weed plants died. Moreover, no new weed germination was observed on the ground surface.

### 3.3. Determination of Pesticide Recovery Rate and Residue After Drip Irrigation Application

As presented in Table 5, for Flumioxazin, across the three matrices, at the spiked levels of 10 and 100 µg/kg, the average recoveries ranged from 78.62% to 97.46%. The relative standard deviations (RSDs) (*n* = 7) were between 4.7% and 7.6%. Regarding Prometryn, its average recoveries across the three matrix species were in the range of 75.80–95.70%, and the relative standard deviations (RSDs) (*n* = 7) fell between 4.2% and 7.9%. For Fluridone, the average recoveries across the three matrices were from 78.92% to 93.46%, and the relative standard deviations (RSDs) (*n* = 7) ranged from 4.8% to 7.2%. These results indicate that the method demonstrated good accuracy and stability and was capable of meeting the requirements for pesticide residue analysis in the cotton field.

As depicted in Table 6, no detectable pesticide residues were identified on cotton leaves 7 days and 16 days after the application of 48% Flumioxazin SC. Conversely, after the application of 42% Fluridone SC, the pesticide residues in the leaves were 167.8 µg/kg and 92.6 µg/kg at 7 days and 16 days post-application, respectively. When 50% Prometryn SC was applied, the pesticide residue in the leaves was 28.7 µg/kg 7 days after application, and this value decreased to 18.6 μg/kg 16 days after application. Concerning pesticide accumulation in cottonseed, only 1.2 µg/kg of pesticide residues were detected in cottonseed after the application of 42% Fluridone SC, while no pesticide residues were detected in cottonseed for the other pesticides. After the application of all three pesticides, pesticide residues were detected in the soil. Among them, the highest amount of pesticide residues, 8.3 µg/kg, was observed for 50% Prometryn SC.

### 3.4. Effect of Drip Irrigation on Cotton Yield

As presented in Table 7, a notable difference existed between the impacts of drip irrigation with herbicides and the water control treatment (CK) on cotton yield. The maximum yield difference per acre reached 51 kg/hm^2^. The yields of 48% Flumioxazin SC at a dosage of 100.8 g a.i./hm^2^ and 50% Prometryn SC at 900 g a.i./hm^2^ exceeded 5550.0 kg/hm^2^. The yield of 42% Fluridone SC at 252 g a.i./hm^2^ was 5442.6 kg/hm^2^, and the cotton yield of 48% Flumioxazin SC at 50.4 g a.i./hm^2^ was 5213.7 kg/hm^2^. With the increase in the application rate of 48% Flumioxazin SC, the yield demonstrated an upward trend. These findings indicated that within a specific concentration range, higher herbicide dosages were more effective in suppressing late-stage weed germination. This suppression ensured the sufficient supply of water and fertilizers to cotton plants, ultimately contributing to an enhancement in cotton yield.

### 3.5. Effect of Drip Irrigation on Cotton Quality

As presented in Table 8, no significant differences were observed in the average length, breaking strength, and elongation at break of the upper half of cotton among the various treatments. These parameters remained within the ranges of 28.33–29.4 mm, 27.43–28.93 cN/tex, and 6.67–6.77%, respectively, showing no significant differences compared to the water control. Notably, the uniformity index of cotton treated with 48% Flumioxazin SC at a dosage of 100.8 g a.i./hm^2^ was significantly different from that of the water control, registering at 80.80%. Moreover, the micronaire value of cotton treated with 48% Flumioxazin SC at 100.8 g a.i./hm^2^ was lower than that of each treatment, and the difference was significant, with a micronaire value of 4.59. All the indexes in this experiment fell within the scope of China’s standard quality indexes for cotton [16].

## 4. Discussion

In the cotton field, the dominant annual broadleaf weeds include *S. nigrum, C. album*, and *C. arvensis*. The efficacy of herbicides applied via drip irrigation was evaluated to determine their control effectiveness against these weed species.

The results indicated that after the application of 50% Prometryn SC and 42% Fluridone SC, a significant control effect was observed on one-year-old broadleaf weeds such as *S. nigrum* and *C. album* during the fruit-setting stage. However, *C. arvensis* remained a challenging weed to control in the cotton field [17]. Additionally, 42% Fluridone SC demonstrated a relatively slow action in suppressing weed growth.

These findings are consistent with the results reported by Zhao et al. (2020) [18] and Guo et al. (2020) [19], who also used drip irrigation to apply these herbicides and found that both agents effectively controlled *S. nigrum* and *C. album*.

The application of 48% Flumioxazin SC led to rapid withering and death of weeds such as *S. nigrum* and *C. album* within 7 days. However, it had no control effect on *C. arvensis*. Post-treatment residue analysis revealed that only pesticide residues were detectable in the soil, and no Flumioxazin was detected in cotton leaves or cottonseeds, suggesting a favorable safety profile for cotton. In contrast, pesticide residues of 50% Prometryn SC and 42% Fluridone SC were detected in cotton leaves at 7 days and 16 days, indicating that the application of these two herbicides poses certain risks to cotton and environmental safety.

Furthermore, compared to the water control group, the application of herbicides via drip irrigation increased cotton yield. Notably, a low concentration of 48% Flumioxazin SC had a limited effect on weed growth. Higher concentrations of 48% Flumioxazin SC exhibited significant weed control efficacy and ultimately increased cotton yield. Therefore, the use of Flumioxazin and Fluridone alone did not result in a reduction in cotton yield [20,21].

The results of this study in 2022 and 2023 indicated that following the drip irrigation on July 25, the growth of *S. nigrum* and *C. album* did not resume after they withered. By August 2, the upper branches and leaves of *S. nigrum* and *C. album* during the fruit-setting stage had died, and new leaves emerged from the lower parts of the plants. Owing to this regrowth, the weeds did not die completely, and notably, no fruit emergence was observed in the subsequent investigation. Therefore, selecting the appropriate period for drip irrigation is of great significance in weed control [22]. Simultaneously, paying attention to the application interval prior to planting may extend the duration of herbicide efficacy in weed control and contribute to maintaining optimal yields [23]. Generally, the earlier the herbicide is applied, the better the control effect; however, this may pose risks to the safe yield and quality of cotton. The experimental investigation demonstrated that the number of newly germinated weeds in the water control treatment increased by approximately 40% within 10 days. In contrast, the number of germinated weeds was significantly suppressed after the application via drip irrigation. Evidently, drip irrigation application not only targeted the emerged weeds but also inhibited the germination of weeds yet to emerge, thus providing a more sustainable control effect. The critical period of weed control is crucial for the effective prevention and control of weeds. Besides the selection of herbicide types, field management practices also play a vital role as key determinants of weed control efficacy. It has been found that the configuration of row spacing, whether single or multiple rows, along with the width of row spacing, significantly impacts the efficacy of weed control. Moreover, cotton cultivated in double-row configurations attains canopy closure earlier than that in single-row arrangements, resulting in increased lint yield [24,25]. Narrower row spacing shortens the duration of competition between weeds and crops, thereby reducing the reliance on herbicide applications and enhancing the natural weed resistance of cotton. Cotton growers can also cut down on weed control costs through more effective weed management timing [26,27].

The technology of water and fertilizer integration has been extensively applied in the Xinjiang cotton region. Research findings indicate that under drip irrigation conditions, soil texture, dripper flow rate, and irrigation volume are the primary determinants influencing water diffusion. The selection of an appropriate dripper flow rate and irrigation volume is pivotal for the distribution of irrigation water within the root zone [28].

In scenarios where the ground surface is uneven, there may be insufficient flow of the herbicide solution, preventing it from reaching the targeted weed-infested areas, which can potentially result in subpar weed control. Simultaneously, studies have revealed that the combination of Flumioxazin with other herbicides effectively reduces the dry weight of weeds. Thus, herbicide combinations are of great significance in surmounting field management challenges and choosing suitable herbicides for diverse field conditions [29,30].

Likewise, in the face of the increasingly intricate field environment of pests and diseases, growers can implement integrated weed and pest management strategies. By choosing compatible mixtures of herbicides and pesticides, it is feasible to cut down on application costs without sacrificing the effectiveness of either pest or weed control measures. Consequently, future research should concentrate on integrating different herbicide combinations or combined pesticide–herbicide formulations with drip irrigation technology to explore their synergistic effects on weed control under field conditions [31]. Notwithstanding the growing adoption of drip irrigation technology, research on its application in the field remains somewhat limited [32]. Given that numerous factors influence the use of drip irrigation for weed, disease, and pest control, a systematic investigation is imperative to optimize this approach and maximize the efficacy of weed control.

## 5. Conclusions

The application of 48% Flumioxazin SC at a rate of 100.8 g a.i./hm^2^ via drip irrigation exhibited a high degree of safety for cotton growth during the boll-forming stage. Simultaneously, it effectively controlled annual broadleaf weeds, such as *S. nigrum* and *C. album*, during the fruit-setting stage. This method also suppressed the germination of weed seeds in the soil and inhibited the emergence of a new generation of weed seeds.

Following the drip irrigation process, pesticide residue analysis showed that no detectable pesticide residues were present in cotton leaves and cottonseeds. Only 6.6 μg/kg of residues were detected in the soil, indicating a minimal environmental footprint. The cotton yield reached 5618.1 kg/hm^2^, and there was no negative impact on the quality of cotton fiber.

Consequently, the results of this study indicate that 48% Flumioxazin SC is a feasible choice for drip-irrigation-based weed management in cotton fields, especially for the control of *S. nigrum* and *C. album*. Nevertheless, further investigations are required to evaluate its applicability under diverse agronomic and environmental conditions. In addition, the intelligent water–herbicide management system requires further refinement. The implementation of this technology should be tailored to specific site conditions to optimize its effectiveness and sustainability within integrated weed control strategies.

## Figures and Tables

**Table 1:** Experimental conditions of field efficacy trials across test sites in 2022 and 2023.

Location	Pre-Sowing Herbicide	Field Conditions	Weed Species (Relative Abundance)
Shihezi University Experimental Field (44°19′27″, 85°58′52″)	33% pendimethalin EC 1485 g a.i./hm^2^	Mulching: Polyethylene film width = 2.25 m Irrigation: Dipper spacing = 0.3 m Herbicide application: - 25 July 2022 and 2023: Chemigation (*S. nigrum* at the blooming stage) - 2 August 2022 and 2023: Herbicide delivered via drip irrigation (*S. nigrum* and *C. album* at the fruit-setting stage)	- *S. nigrum* + *C. album* (70% total weed density) - *C. arvensis* (20%)

**Table 2:** Drip irrigation was used to test herbicides.

Herbicides	Active Ingredient Dosage (g a.i./hm^2^)	Production Enterprise
48% Flumioxazin SC	50.4	Jiangsu Yongan Chemical Co., Ltd.
48% Flumioxazin SC	100.8	Jiangsu Yongan Chemical Co., Ltd.
42% Fluridone SC	252	Max (Rudong) Chemicals Co., Ltd. (Rudong, China)
50% Prometryn SC	900	Max (Rudong) Chemicals Co., Ltd.

**Table 3:** Effects of herbicides on cotton after drip irrigation in 2022 and 2023.

Treatments	Active Gradient (g a.i./hm^2^)	Phytotoxicity Grade	Characteristics of Phytotoxicity
48% Flumioxazin SC	50.4	2	10% of upper leaves exhibited slight reddening; lower leaves remained unaffected.
48% Flumioxazin SC	100.8	2	20% of upper leaf veins and lamina displayed red discoloration.
42% Fluridone SC	252	2	20% of upper leaves showed reddening with downward curling
50% Prometryn SC	900	2	40% of upper leaf veins developed mild reddening
Clear water control (CK)	0	1	No observable symptoms

Phytotoxicity grading scale: 1 = no visible symptoms, 2 = slight-to-moderate symptoms. The same criteria for phytotoxic symptoms have been applied in 2022 and 2023.

**Table 4:** Control effect of drip irrigation herbicide on weeds in cotton field in 2022 and 2023.

Herbicides/(g a.i./hm^2^)		Before Application	7 Days After Application		16 Days After Application	
		Weed Count	Weed Count	Control Effect/%	Weed Count	Control Effect/%
48% Flumioxazin SC	50.4	48	16	66.67 a	17	64.58 ab
48% Flumioxazin SC	100.8	64	20	68.75 a	19	70.31 a
42% Fluridone SC	252	42	42	0 c	13	69.05 a
50% Prometryn SC	900	36	14	61.11 b	13	63.89 b

Note: Different lowercase letters in the same column indicate a significant difference at *p* < 0.05. All the data pooled over 2022 and 2023.

**Table 5:** Average recovery and relative standard deviation of herbicides in different substrates in 2022 and 2023.

Matrices	Addition Level/(µg/kg)	Flumioxazin		Prometryn		Fluridone	
		Average Recovery/%	RSD/%	Average Recovery/%	RSD/%	Average Recovery/%	RSD/%
Cotton Leaves	10	78.62	6.4	75.80	7.9	78.92	6.5
	100	93.43	4.8	95.01	4.2	92.98	4.8
Cotton seed	10	82.74	7.6	76.13	6.8	82.48	7.2
	100	97.46	5.2	95.70	4.5	91.42	4.8
Soil	10	90.11	6.8	88.20	7.5	93.46	6.9
	100	92.40	4.7	91.20	4.8	89.43	4.8

**Table 6:** Residual amount of each substrate after drip irrigation application of herbicide in 2022 and 2023.

Herbicides/(g a.i./hm^2^)		Pesticide Residues/(µg/kg)			
		Cotton Leaves		Cottonseed	Soil
		7 d	16 d		
48% Flumioxazin SC	50.4	ND	ND	ND	4.8
48% Flumioxazin SC	100.8	ND	ND	ND	6.6
42% Fluridone SC	252	167.8	92.6	1.2	7.2
50% Prometryn SC	900	28.7	18.6	ND	8.3

Note: ND (No detection) means that no residue was detected or there was residue display, but no specific peak shape. All the data pooled over 2022 and 2023.

**Table 7:** Effect of herbicide application on yield under drip irrigation in 2022 and 2023.

Treatments	Active Ingredient Dosage (g a.i./hm^2^)	Cotton Production (kg/hm^2^)
48% Flumioxazin SC	50.4	5213.7 bB
48% Flumioxazin SC	100.8	5618.1 aA
42% FluridoneSC	252	5442.6 bA
50% Prometryn SC	900	5578.4 aA
Clear water control (CK)	/	4853.6 cB

Note: Different lowercase letters in the same column indicate significant differences at *p* < 0.05, and different uppercase letters indicate significant differences at *p* < 0.01.

**Table 8:** Effect of drip irrigation application of herbicides on cotton quality in 2022 and 2023.

Herbicides/(g a.i./hm^2^)		Quality Index				
		Average Length of Upper Half (mm)	Uniformity Index (%)	Breaking Strength (cN/tex)	Elongation (%)	Micronaire
48% Flumioxazin SC	50.4	28.67 aA	82.23 abA	27.43 aA	6.67 aA	4.59 cB
48% Flumioxazin SC	100.8	28.40 aA	80.80 bA	27.47 aA	6.67 aA	5.04 aA
42% Fluridone SC	252	29.40 aA	83.37 abA	28.93 aA	6.77 aA	4.84 bA
50% Prometryn SC	900	28.33 aA	82.70 abA	27.83 aA	6.67 aA	4.82 bA
Clear water control (CK)	/	29.23 aA	83.53 aA	27.87 aA	6.77 aA	4.87 bA

Note: Different lowercase letters in the same column indicate significant differences at *p* < 0.05, and different uppercase letters indicate the significant differences at *p* < 0.01. All the data pooled over 2022 and 2023.

## Data Availability

The datasets supporting the results presented in this manuscript are included within the article (and its Supplementary Materials).

## Supplementary Materials

The following supporting information can be downloaded at: https://www.mdpi.com/article/10.3390/plants14111589/s1, Figure S1. Cotton planting distribution of one plastic film (2.05 m). Table S1. Agricultural technology operation in the process of field experiment. Table S2. Weather conditions during the test in 2022. Table S3. Weather conditions during the test in 2023. Table S4. Drip irrigation application of herbicides on cotton quality in 2022 and 2023. Table S5. Yield of herbicide application under drip irrigation in 2022 and 2023.
