# Supplementary material for: The Broadleaf Weeds Control Efficiency of Drip Irrigation Herbicides in Cotton Fields and the Cotton Safety Assessment
Source: Plants (Basel). 2025 May 23;14(11):1589. doi: 10.3390/plants14111589 (PMC12158021; doi:10.3390/plants14111589)
Supplement: Supplementary file 1 [file plants-14-01589-s001.zip › plants-3579685-supplementary.pdf]

Supplementary Material

The Control Efficiency of Drip Irrigation Herbicides on Broadleaf Weeds in Cotton Fields and the Safety Evaluation of Cotton

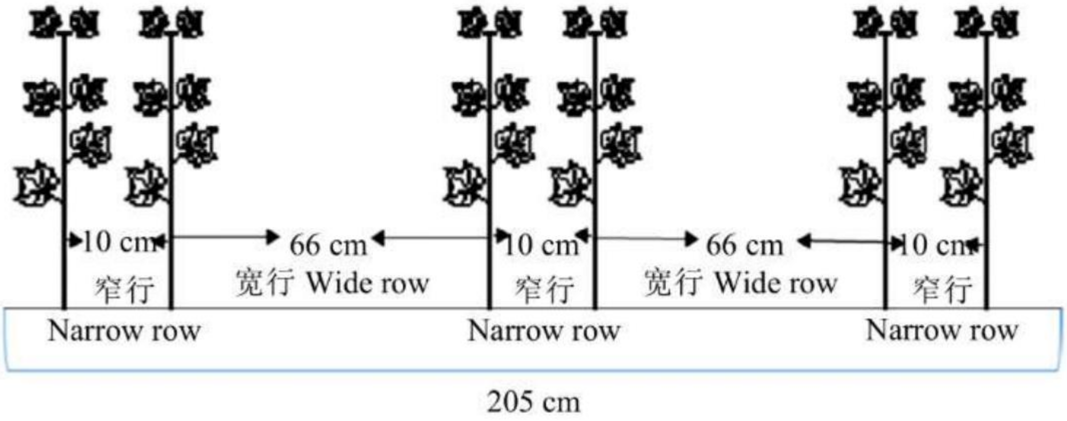

Figure S1. Cotton planting distribution of one plastic film (2.05 m)

Table S1. Agricultural technology operation in the process of field experiment

Management Type	Management Description
Tillage Management	April 20 in 2022 and April 19 in 2023
Irrigation	-April 22 and April 24: Two times of drip irrigation seedling water (each time 12 m^3)<br>-June 10 (40 m^3), June 20 (30 m^3), June 30 (30 m^3), July 7 (30 m^3), July 15 (30 m^3), July 22 (30 m^3), July 30 (30 m^3), August 7 (30 m^3), August 15 (30 m^3), August 20 (30 m^3), August 26 (30 m^3)
Fertilization	-June 10: urea (60 kg/hm^2);<br>-June 20: urea (90 kg/hm^2) + K_2SO_4(30 kg/hm^2) + monoammonium phosphate (30 kg/hm^2)<br>-June 30 and July 7: urea (90 kg/hm^2) + K_2SO_4 (45 kg/hm^2) + monoammonium phosphate (30 kg/hm^2)<br>-July 15 and July 22: urea (90 kg/hm^2) + K_2SO_4 (60 kg/hm^2) + monoammonium phosphate (30 kg/hm^2)<br>-July 30: urea (75 kg/hm^2) + K_2SO_4 (60 kg/hm^2) + monoammonium phosphate (30 kg/hm^2)<br>-August 7 and August 15: urea (60 kg/hm^2) + K_2SO_4 (45 kg/hm^2) + monoammonium phosphate (30 kg/hm^2)<br>-August 20: urea (22.5 kg/hm^2) + K_2SO_4 (30 kg/hm^2) + monoammonium phosphate (30 kg/hm^2)

	-August 26: urea (22.5 kg/hm^2) + K_2SO_4 (30 kg/hm^2) + monoammonium phosphate (15 kg/hm^2)
Other Pesticide Use	-May 10: Imidacloprid soluble liquid agent (150mL/hm^2)<br>-June 12, June 22, July 3, July 10, July 15, July 30: 25% Mepiquat chloride aqueous solution (150mL/hm^2)<br>-July 10: 25% Prime + 250E (900mL/hm^2)<br>-September 5: Diuron. Thidiazuron Suspension Concentrate (150mL/hm^2)
Harvest	October 3,2022 and 2023

Note: Irrigation, fertilizer and other pesticide use are already in line for 2022 and 2023.

Table S2. Weather conditions during the test in 2022

Date	Temperature (℃)		precipitation amount (mm)	wind scale	weather conditions	note
	minimum	maximum				
April 20,2022	11	23	-	North wind two-level	Cloudy to situation	Sowing cotton
April 21,2022	10	22	-	North-west wind two-level	Cloudy to clear	
April 22,2022	10	27	-	North-west wind two-level	Cloudy to clear	
April 23,2022	12	25	-	North-east wind two-level	Clear	
April 24,2022	9	19	0.6	North-east wind three-level	Cloudy to light rain	
April 25,2022	7	17	1.2	North-east wind three-level	Cloudy to light rain	
April 26,2022	4	15	-	North-west wind two-level	Fog to clear	
April 27,2022	9	18	-	North wind two-level	Situation	
April 28,2022	7	21	-	North-west wind three-level	Cloudy to clear	
April 29,2022	6	22	-	Southeaster three-level	Clear	
April 30,2022	9	29	-	East wind three-level	Clear	
May 1,2022	11	29	-	Southeaster two-level	Clear to situation	

May 2,2022	15	32	-	East wind two-level	Clear	
May 3,2022	15	33	-	North-east wind two-level	Clear to situation	
May 4,2022	15	28	-	North-east wind three-level	Cloudy to situation	
May 5,2022	15	29	0.8	South-west wind two-level	Cloudy to light rain	
May 6,2022	13	19	5.0	West wind three-level	Moderate rain to clear	
May 7,2022	17	28	-	North-east wind two-level	Cloudy to clear	
May 8,2022	14	29	-	North-west wind three-level	Cloudy to clear	
May 9,2022	13	23	-	East wind four-level	Cloudy to clear	
May 10,2022	14	27	-	East wind three-level	Cloudy to situation	
May 11,2022	14	25	-	North-west wind three-level	Cloudy to situation	
May 12,2022	12	23	-	West wind two-level	Cloudy to situation	
May 13,2022	14	27	-	Southeaster three-level	Cloudy to situation	
May 14,2022	16	30	-	East wind three-level	Cloudy to situation	
May 15,2022	14	29	0.9	West wind three-level	Cloudy to light rain	
May 16,2022	14	25	0.7	South-west wind three-level	Light rain to situation	
May 17,2022	14	29	-	East wind two-level	Cloudy to clear	
May 18,2022	16	31	-	Southeaster two-level	Clear	
May 19,2022	18	33	-	West wind two-level	Cloudy to situation	

May 20,2022	16	30	-	West wind three-level	Cloudy to clear	
May 21,2022	17	31	-	East wind three-level	Cloudy to situation	
May 22,2022	19	32	-	West wind three-level	Cloudy to situation	
May 23,2022	17	30	-	West wind three-level	Cloudy to clear	
May 24,2022	18	31	-	North-east wind two-level	Cloudy to situation	
May 25,2022	23	34	-	North-west wind two-level	Cloudy to situation	
May 26,2022	20	33	-	West wind three-level	Cloudy to clear	
May 27,2022	21	32	-	West wind four-level	Cloudy to clear	
May 28,2022	20	34	-	West wind four-level	Clear	
May 29,2022	17	30	-	North-west wind three-level	Clear to situation	
May 30,2022	18	28	-	North-west wind three-level	Cloudy	
May 31,2022	16	27	0.2	West wind three-level	Cloudy to light rain	
June 1,2022	15	24	-	South-west wind three-level	Cloudy to clear	
June 2,2022	11	22	-	North-east wind three-level	Situation to clear	
June 3,2022	13	24	-	North-east wind two-level	Cloudy	
June 4,2022	14	27	-	North-east wind two-level	Cloudy to clear	
June 5,2022	16	27	-	West wind two-level	Clear	
June 6,2022	16	28	-	North-east wind three-	Cloudy to situation	

				level		
June 7,2022	17	29	-	North-east wind two-level	Cloudy to clear	
June 8,2022	16	30	-	North-east wind two-level	Clear	
June 9,2022	19	34	-	Southeaster two-level	Cloudy to situation	
June 10,2022	17	33	-	South-west wind two-level	Cloudy to clear	
June 11,2022	19	27	-	Southeaster two-level	Clear	
June 12,2022	23	39	-	South wind two-level	Cloudy	
June 13,2022	23	39	-	South-west wind two-level	Clear to situation	
June 14,2022	19	33	-	West wind four-level	Cloudy to clear	
June 15,2022	19	31	-	South wind two-level	Cloudy to situation	
June 16,2022	19	24	-	South-west wind three-level	Cloudy to situation	
June 17,2022	18	32	-	North-west wind two-level	Cloudy to clear	
June 18,2022	18	30	-	North-east wind two-level	Clear to situation	
June 19,2022	20	29	-	South-west wind three-level	Cloudy to situation	
June 20,2022	17	33	-	South-west wind two-level	Cloudy to clear	
June 21,2022	19	35	-	North-west wind two-level	Clear	
June 22,2022	18	37	-	East wind three-level	Cloudy to clear	

June 23,2022	23	39	-	East wind two-level	Clear	
June 24,2022	22	32	-	West wind four-level	Cloudy to situation	
June 25,2022	22	32	-	West wind four-level	Cloudy to clear	
June 26,2022	21	35	-	North-west wind two-level	Clear	
June 27,2022	25	37	-	Southeaster two-level	Cloudy to clear	
June 28,2022	21	38	-	West wind three-level	Cloudy to clear	
June 29,2022	21	34	-	North-east wind two-level	Clear	
June 30,2022	21	38	-	North-east wind three-level	Cloudy to situation	
July 1,2022	20	33	-	North wind three-level	Cloudy to clear	
July 2,2022	16	30	-	North-east wind two-level	Cloudy to clear	
July 3,2022	17	31	-	Southeaster three-level	Cloudy to clear	
July 4,2022	19	33	-	East wind two-level	Cloudy to clear	
July 5,2022	17	28	0.5	North-west wind three-level	Cloudy to light rain	
July 6,2022	13	26	-	Southeaster two-level	Cloudy to situation	
July 7,2022	17	28	-	Southeaster two-level	Cloudy to clear	
July 8,2022	16	30	-	East wind three-level	Clear to situation	
July 9,2022	16	27	-	North-west wind three-level	Cloudy to clear	
July 10,2022	17	30	-	North-east wind two-level	Clear	

July 11,2022	19	32	-	North-west wind three-level	Cloudy to situation	
July 12,2022	18	28	-	South-west wind three-level	Cloudy to situation	
July 13,2022	19	30	-	West wind four-level	Cloudy to clear	
July 14,2022	16	31	-	North-west wind three-level	Cloudy to clear	
July 15,2022	18	30	-	North-east wind two-level	Clear to situation	
July 16,2022	19	33	-	East wind two-level	Cloudy to clear	
July 17,2022	19	36	-	South-west wind two-level	Cloudy to clear	
July 18,2022	18	34	-	North-west wind three-level	Cloudy to situation	
July 19,2022	19	27	-	North-west wind two-level	Cloudy to clear	
July 20,2022	16	29	-	North-west wind four-level	Cloudy to clear	
July 21,2022	15	32	-	East wind two-level	Clear	
July 22,2022	20	34	-	North-east wind two-level	Clear	
July 23,2022	18	37	-	North-east wind three-level	Clear	
July 24,2022	19	31	-	North-east wind two-level	Clear to situation	
July 25,2022	20	34	-	North-west wind two-level	Cloudy to clear	Drip Irrigation Herbicides
July 26,2022	18	32	-	North-east wind two-level	Cloudy to clear	
July 27,2022	20	34	-	North-west wind two-level	Clear to situation	
July 28,2022	21	36	-	North-west wind two-level	Cloudy to situation	
July 29,2022	21	35	-	South-west wind three-level	Cloudy to clear	

July 30,2022	20	34	-	West wind three-level	Cloudy to clear	
July 31,2022	18	30	-	West wind two-level	Cloudy to clear	
August 1,2022	20	31	-	Southeaster two-level	Cloudy to clear	
August 2,2022	19	32	-	North-west wind two-level	Cloudy to clear	Drip Irrigation Herbicides
August 3,2022	21	33	-	North-east wind two-level	Fog to cloudy	
August 4,2022	20	32	-	North-west wind three-level	Cloudy to clear	
August 5,2022	20	31	-	North-west wind two-level	Cloudy	
August 6,2022	20	32	-	Southeaster two-level	Cloudy to clear	
August 7,2022	20	33	-	Southeaster two-level	Clear	
August 8,2022	18	33	0.7	West wind two-level	Cloudy to light rain	
August 9,2022	19	32	-	East wind three-level	Clear to cloudy	
August 10,2022	18	30	5.4	North-west wind two-level	Cloudy to moderate rain	
August 11,2022	19	29	5.9	East wind two-level	Cloudy to moderate rain	
August 12,2022	15	25	0.3	South-west wind two-level	Light rain to clear	
August 13,2022	17	30	-	North-west wind two-level	Clear	
August 14,2022	15	32	-	North-east wind two-level	Clear	
August 15,2022	18	34	-	East wind three-level	Cloudy to clear	
August 16,2022	16	29	-	North-east wind two-	Cloudy to clear	

				level		
August 17,2022	14	26	-	North-east wind three-level	Cloudy to clear	
August 18,2022	16	26	-	North-west wind two-level	Clear	
August 19,2022	13	27	-	North-east wind two-level	Clear	
August 20,2022	14	22	-	North-east wind three-level	Cloudy to clear	
August 21,2022	14	25	-	North-east wind two-level	Cloudy to situation	
August 22,2022	9	18	0.6	South-west wind three-level	Light rain to situation	
August 23,2022	8	24	-	East wind three-level	Cloudy to clear	
August 24,2022	13	27	-	East wind two-level	Clear to situation	
August 25,2022	15	23	-	North-east wind one-level	Cloudy to situation	
August 26,2022	17	25	-	South-west wind two-level	Cloudy to situation	
August 27,2022	14	29	-	North-west wind two-level	Cloudy to clear	
August 28,2022	12	29	-	West wind two-level	Clear	
August 29,2022	13	31	-	North-east wind two-level	Clear	
August 30,2022	15	28	-	North-east wind two-level	Cloudy to clear	
August 31,2022	16	29	-	South-west wind two-level	Clear	

September 1,2022	17	32	-	West wind two-level	Clear	
September 2,2022	17	33	-	West wind three-level	Clear	
September 3,2022	16	32	-	West wind three-level	Clear	
September 4,2022	14	32	-	North-west wind two-level	Clear	
September 5,2022	15	33	-	East wind two-level	Clear	
September 6,2022	17	33	-	West wind two-level	Clear	
September 7,2022	17	36	-	Southeaster two-level	Clear	
September 8,2022	18	38	-	Southeaster two-level	Clear	
September 9,2022	14	31	-	North wind three-level	Cloudy to situation	
September 10,2022	13	29	-	East wind two-level	Cloudy to clear	
September 11,2022	12	29	-	North-east wind two-level	Cloudy to situation	
September 12,2022	10	18	1.2	North-west wind three-level	Light rain	
September 13,2022	7	16	0.7	North-east wind two-level	Light rain to clear	
September 14,2022	10	23	-	North-east wind two-level	Fog to situation	
September 15,2022	5	20	-	North-west wind two-level	Cloudy to clear	
September 16,2022	6	21	-	North-east wind three-level	Clear	
September 17,2022	7	24	-	North-east wind two-level	Clear	
September 18,2022	8	25	-	East wind two-level	Cloudy to clear	

September 19,2022	10	25	-	North-east wind two-level	Clear to situation	
September 20,2022	10	27	-	North-east wind one-level	Cloudy to clear	
September 21,2022	12	29	-	East wind two-level	Clear	
September 22,2022	14	29	-	North-east wind two-level	Cloudy to situation	
September 23,2022	13	30	-	North-west wind two-level	Cloudy	
September 24,2022	12	30	-	Southeaster two-level	Clear	
September 25,2022	13	30	-	West wind one-level	Cloudy to clear	
September 26,2022	13	31	-	West wind two-level	Clear to cloudy	
September 27,2022	11	30	-	North-east wind three-level	Cloudy to clear	
September 28,2022	10	18	-	North-east wind two-level	Cloudy to clear	
September 29,2022	10	21	0.6	North-east wind two-level	Cloudy to light rain	
September 30,2022	0	9	-	North-east wind three-level	Cloudy to sleet	
October 1,2022	3	14		West wind two-level	Clear	
October 2,2022	5	18		Southeaster two-level	Clear	
October 3,2022	6	20		North-east wind one-level	Clear	Harvest

Table S3. Weather conditions during the test in 2023

Date	Temperature (℃)		precipitation amount (mm)	wind scale	weather conditions	note
	minimum	maximum				
April 19,2023	0	11	-	North-east wind three-level	Cloudy	Sowing cotton
April 20,2023	0	13	-	North-east wind three-level	Fog to clear	
April 21,2023	3	15	-	North-east wind three-level	Cloudy to situation	
April 22,2023	4	15	-	North-east wind two-level	Cloudy to clear	
April 23,2023	4	17	-	North-east wind one-level	Cloudy to clear	
April 24,2023	9	22	-	North-east wind two-level	Clear to situation	
April 25,2023	9	19	-	West wind three-level	Cloudy to situation	
April 26,2023	9	20	-	North-west wind four-level	Cloudy to clear	
April 27,2023	8	23	-	West wind three-level	Clear	
April 28,2023	7	26	-	East wind two-level	Clear	
April 29,2023	14	28	-	North-east wind two-level	Clear	
April 30,2023	15	29	-	West wind three-level	Cloudy to situation	
May 1,2023	7	19	1.0	North-west wind two-level	Light rain to situation	
May 2,2023	5	10	0.8	North wind two-level	Cloudy to light rain	
May 3,2023	3	11	0.3	North-west wind three-level	Cloudy to light rain	
May 4,2023	3	11	-	North-west wind three-level	Cloudy	
May 5,2023	3	13	-	North-west wind three-level	Situation to cloudy	
May 6,2023	6	15	-	North-east	Cloudy to	

				wind two-level	situation	
May 7,2023	4	15	-	North-west wind two-level	Cloudy to situation	
May 8,2023	10	20	-	North-west wind two-level	Cloudy to clear	
May 9,2023	13	26	-	West wind three-level	Cloudy to clear	
May 10,2023	11	28	-	North-west wind one-level	Clear	
May 11,2023	13	31	-	East wind two-level	Cloudy to situation	
May 12,2023	19	33	-	North-west wind two-level	Cloudy to situation	
May 13,2023	17	32	-	North-west wind two-level	Cloudy to clear	
May 14,2023	16	31	-	North-west wind two-level	Cloudy to situation	
May 15,2023	14	21	-	Southeaster two-level	Cloudy to situation	
May 16,2023	13	21	-	North-east wind two-level	Cloudy	
May 17,2023	13	27	-	North-east wind one-level	Cloudy to clear	
May 18,2023	14	28	1.4	North-east wind two-level	Clear to light rain	
May 19,2023	13	24	-	East wind two-level	Cloudy to clear	
May 20,2023	14	26	1.1	North wind one-level	Light rain to situation	
May 21,2023	12	26	-	North wind one-level	Cloudy to clear	
May 22,2023	14	23	-	North-west wind two-level	Cloudy to clear	

May 23,2023	14	25	-	North-west wind four-level	Clear	
May 24,2023	15	27	-	North-west wind three-level	Cloudy to situation	
May 25,2023	15	22	-	West wind three-level	Cloudy to clear	
May 26,2023	13	27	-	North-west wind four-level	Clear	
May 27,2023	16	27	-	North-east wind one-level	Clear to situation	
May 28,2023	13	25	0.5	North-west wind three-level	Cloudy to light rain	
May 29,2023	13	21	-	West wind three-level	Cloudy to clear	
May 30,2023	13	24	-	North-west wind one-level	Clear to situation	
May 31,2023	12	26	-	North-west wind two-level	Cloudy to clear	
June 1,2023	14	25	-	North-west wind one-level	Cloudy to situation	
June 2,2023	13	27	-	North-east wind two-level	Cloudy to clear	
June 3,2023	14	30	-	North-east wind two-level	Cloudy to clear	
June 4,2023	15	32	-	East wind three-level	Cloudy to clear	
June 5,2023	16	35	-	East wind two-level	Clear	
June 6,2023	17	37	-	North-east wind two-level	Situation to clear	
June 7,2023	21	38	-	North-east wind one-level	Cloudy to situation	

June 8,2023	22	36	-	West wind three-level	Cloudy to clear	
June 9,2023	19	38	-	North-west wind two-level	Clear	
June 10,2023	23	39	-	North-east wind one-level	Clear	
June 11,2023	21	37	-	North-east wind one-level	Cloudy to situation	
June 12,2023	18	29	-	North-west wind three-level	Cloudy to clear	
June 13,2023	18	32	-	North-east wind three-level	Cloudy to clear	
June 14,2023	20	31	-	North wind two-level	Cloudy to situation	
June 15,2023	18	27	-	Southeaster two-level	Cloudy to situation	
June 16,2023	16	28	-	North-west wind one-level	Cloudy to clear	
June 17,2023	18	31	-	North-west wind two-level	Clear	
June 18,2023	20	34	-	North-east wind two-level	Clear to cloudy	
June 19,2023	23	36	-	Southeaster three-level	Cloudy to situation	
June 20,2023	21	35	-	North-east wind two-level	Cloudy	
June 21,2023	20	32	-	North-east wind two-level	Cloudy to situation	
June 22,2023	18	32	-	East wind three-level	Cloudy to clear	
June 23,2023	18	31	-	North-east wind one-level	Cloudy to situation	

June 24,2023	15	25	0.9	North-west wind two-level	Light rain to clear	
June 25,2023	17	25	-	North-east wind two-level	Cloudy to situation	
June 26,2023	12	20	1.5	East wind two-level	Light rain to clear	
June 27,2023	15	29	-	East wind three-level	Clear to situation	
June 28,2023	18	33	-	North-east wind two-level	Cloudy to clear	
June 29,2023	15	29	0.4	North-west wind three-level	Cloudy to light rain	
June 30,2023	15	26	-	North-west wind one-level	Cloudy to situation	
July 1,2023	15	26	-	South-west wind two-level	Cloudy to clear	
July 2,2023	13	27	-	North-west wind two-level	Cloudy to clear	
July 3,2023	15	32	-	North-east wind two-level	Clear to situation	
July 4,2023	17	34	-	North-west wind three-level	Cloudy to clear	
July 5,2023	17	32	-	North-west wind two-level	Cloudy to clear	
July 6,2023	20	32	-	North-east wind two-level	Cloudy to clear	
July 7,2023	20	32	-	East wind two-level	Cloudy to situation	
July 8,2023	18	25	0.2	South-west wind two-level	Light rain to situation	
July 9,2023	19	28	-	South-west wind two-	Cloudy	

				level		
July 10,2023	18	30	-	South-west wind three-level	Situation to clear	
July 11,2023	18	29	-	North-west wind three-level	Clear	
July 12,2023	15	32	-	North-west wind three-level	Clear	
July 13,2023	17	35	-	East wind two-level	Clear	
July 14,2023	20	38	-	North-east wind two-level	Situation to clear	
July 15,2023	23	39	-	North-east wind two-level	Cloudy to clear	
July 16,2023	22	36	-	North-west wind three-level	Cloudy to situation	
July 17,2023	20	33	-	North-west wind three-level	Situation	
July 18,2023	20	36	-	North-east wind one-level	Cloudy to clear	
July 19,2023	21	38	-	North-east wind two-level	Cloudy to clear	
July 20,2023	21	33	-	North-west wind three-level	Situation to clear	
July 21,2023	23	34	-	North-east wind two-level	Cloudy to situation	
July 22,2023	19	35	-	North-west wind three-level	Situation to clear	
July 23,2023	21	37	-	North-east wind two-level	Situation to clear	
July 24,2023	22	34	-	North-west wind three-level	Situation to clear	
July 25,2023	19	35	-	North-east wind two-level	Cloudy to clear	Drip Irrigation Herbicides
July 26,2023	19	38	-	North-east wind three-level	Situation to clear	
July 27,2023	26	39	-	East wind	Cloudy	

				three-level		
July 28,2023	20	34	-	South-west wind two-level	Situation to clear	
July 29,2023	17	28	-	Southeaster two-level	Situation	
July 30,2023	17	31	-	East wind three-level	Cloudy to clear	
July 31,2023	19	35		North-east wind two-level	Situation	
August 1,2023	22	35	-	South-west wind two-level	Situation to clear	
August 2,2023	21	36	-	North-west wind one-level	Cloudy to situation	Drip Irrigation Herbicides
August 3,2023	18	31	-	Southeaster two-level	Situation to clear	
August 4,2023	20	35	-	North-west wind one-level	Clear	
August 5,2023	19	35	-	North-west wind two-level	Cloudy to clear	
August 6,2023	22	37	-	North-east wind one-level	Cloudy to clear	
August 7,2023	19	36	0.3	North-west wind three-level	Situation to light rain	
August 8,2023	16	23	-	North-west wind three-level	Situation to clear	
August 9,2023	18	32	-	North-east wind two-level	Fog to clear	
August 10,2023	19	33	-	North-east wind three-level	Clear to cloudy	
August 11,2023	18	36	0.2	North-east wind three-level	Situation to light rain	
August 12,2023	15	27	10.7	East wind three-level	Situation to hard rain	
August 13,2023	14	27	-	North-east	Situation	

				wind three-level		
August 14,2023	11	18	1.3	North-west wind two-level	Light rain to situation	
August 15,2023	12	23	-	North-east wind one-level	Situation to clear	
August 16,2023	14	27	-	East wind three-level	Clear	
August 17,2023	15	29	-	East wind two-level	Situation to clear	
August 18,2023	16	30	-	East wind three-level	Cloudy to clear	
August 19,2023	15	32	-	North-west wind one-level	Cloudy to clear	
August 20,2023	17	31	-	North-east wind one-level	Clear	
August 21,2023	17	32	-	North-west wind two-level	Cloudy to clear	
August 22,2023	20	33	-	North-east wind two-level	Situation	
August 23,2023	16	31	-	North-west wind one-level	Situation to clear	
August 24,2023	16	32	-	North-east wind one-level	Cloudy to clear	
August 25,2023	16	35	-	East wind one-level	Clear to situation	
August 26,2023	18	36	-	North-east wind three-level	Cloudy	
August 27,2023	19	34	-	North-west wind one-level	Cloudy to situation	
August 28,2023	17	30	0.3	West wind five-level	Cloudy to light rain	
August 29,2023	13	25	0.5	North-west wind three-	Situation to light	

				level	rain	
August 30,2023	15	29	-	North-east wind one-level	Cloudy to clear	
August 31,2023	16	33	-	North-east wind three-level	Cloudy to clear	
September 1,2023	16	29	-	North-east wind two-level	Situation	
September 2,2023	15	28	-	North-east wind three-level	Cloudy to clear	
September 3,2023	16	31	0.7	North-east wind two-level	Cloudy to light rain	
September 4,2023	14	27	0.5	North-west wind four-level	Situation to light rain	
September 5,2023	10	21	0.5	South-west wind three-level	Light rain to clear	
September 6,2023	9	26	-	North-east wind two-level	Clear to situation	
September 7,2023	10	28	-	North-east wind two-level	Cloudy to clear	
September 8,2023	12	29	-	North-east wind two-level	Situation to cloudy	
September 9,2023	13	29	-	North-east wind two-level	Cloudy to situation	
September 10,2023	12	31	-	North-east wind two-level	Situation to clear	
September 11,2023	12	30	-	North-east wind one-level	Situation to clear	
September 12,2023	13	30	0.2	North-west wind four-level	Cloudy to light rain	

September 13,2023	11	23	0.5	Southeasterone-level	Light rain to situation	
September 14,2023	10	21	6.0	North-east wind two-level	Situation to moderate rain	
September 15,2023	9	16	1.4	North-east wind one-level	Light rain	
September 16,2023	7	20	1.6	North-west wind two-level	Situation to light rain	
September 17,2023	8	20	-	North wind one-level	Situation to clear	
September 18,2023	8	23	-	East wind three-level	Situation to clear	
September 19,2023	9	25	-	East wind three-level	Situation to clear	
September 20,2023	11	25	0.8	North-east wind two-level	Situation to light rain	
September 21,2023	6	20	-	North-west wind two-level	Situation	
September 22,2023	10	22	-	North-west wind two-level	Cloudy to situation	
September 23,2023	6	21	-	West wind three-level	Situation	
September 24,2023	6	11	11.6	North-west wind two-level	Hard rain to light rain	
September 25,2023	3	15	-	Southeasterone-level	Situation to clear	
September 26,2023	7	22	-	West wind two-level	Clear	
September 27,2023	4	20	-	North-east wind one-level	Clear	
September 28,2023	6	21	-	North-east wind three-level	Clear	
September 29,2023	7	22	-	North-east wind two-	Cloudy to clear	

				level		
September 30,2023	9	23	-	North-west wind one-level	Cloudy to clear	
October 1,2023	6	22	-	East wind two-level	Cloudy	
October 2,2023	6	23	-	East wind three-level	Clear to cloudy	
October 3,2023	8	23	-	North-east wind two-level	Cloudy to clear	Harvest

Table S4. Drip irrigation application of herbicides on cotton quality in 2022 and 2023.

Sample Number (Survey Year + M + Label)	Herbicides	Average Length of Upper Half (mm)	Uniformity Index (%)	Breaking Strength (cN/tex)	Elongation (%)	Micronaire
2022-M-01	50.4 g a.i./hm^2 48% Flumioxazin SC	29.24	82.90	27.50	6.70	4.60
2022-M-02	50.4 g a.i./hm^2 48% Flumioxazin SC	28.03	80.50	26.50	6.60	4.61
2022-M-03	50.4 g a.i./hm^2 48% Flumioxazin SC	28.84	81.40	28.10	6.70	4.57
2023-M-01	50.4 g a.i./hm^2 48% Flumioxazin SC	28.97	83.50	27.20	6.60	4.58
2023-M-02	50.4 g a.i./hm^2 48% Flumioxazin SC	28.49	82.30	27.20	6.70	4.58
2023-M-03	50.4 g a.i./hm^2 48% Flumioxazin SC	28.46	82.70	28.10	6.70	4.59
2022-M-04	100.8 g a.i./hm^2 48% Flumioxazin SC	28.15	82.00	26.40	6.70	5.03
2022-M-05	100.8 g a.i./hm^2 48% Flumioxazin SC	28.16	82.50	27.00	6.60	4.94
2022-M-06	100.8 g a.i./hm^2 48% Flumioxazin SC	28.76	82.20	29.00	6.70	5.14

2023-M-04	100.8 g a.i./hm^2 48% Flumioxazin SC	28.17	79.50	26.50	6.80	5.04
2023-M-05	100.8 g a.i./hm^2 48% Flumioxazin SC	28.18	78.00	28.80	6.80	5.05
2023-M-06	100.8 g a.i./hm^2 48% Flumioxazin SC	28.98	80.60	27.10	6.80	5.04
2022-M-07	252 g a.i./hm^2 42% Fluridone SC	29.87	84.00	29.30	6.80	4.79
2022-M-08	252 g a.i./hm^2 42% Fluridone SC	28.91	82.90	28.00	6.70	4.88
2022-M-09	252 g a.i./hm^2 42% Fluridone SC	29.28	83.16	29.50	6.80	4.84
2023-M-07	252 g a.i./hm^2 42% Fluridone SC	29.30	83.45	29.25	6.79	4.80
2023-M-08	252 g a.i./hm^2 42% Fluridone SC	29.29	83.25	28.15	6.75	4.85
2023-M-09	252 g a.i./hm^2 42% Fluridone SC	29.66	83.48	29.38	6.78	4.88
2022-M-10	900 g a.i./hm^2 50% Prometryn SC	28.08	82.80	28.00	6.70	4.77
2022-M-11	900 g a.i./hm^2 50% Prometryn SC	28.85	82.60	28.90	6.70	4.77
2022-M-12	900 g a.i./hm^2 50% Prometryn SC	28.00	82.70	26.72	6.60	4.93
2023-M-10	900 g a.i./hm^2 50% Prometryn SC	28.10	82.75	27.85	6.67	4.83
2023-M-11	900 g a.i./hm^2 50% Prometryn SC	28.15	82.70	27.90	6.68	4.80
2023-M-12	900 g a.i./hm^2 50% Prometryn SC	28.80	82.65	27.79	6.67	4.82

2022-M-13	Clear Water Control	30.70	85.70	29.10	6.80	4.89
2022-M-14	Clear Water Control	27.85	81.50	26.90	6.70	4.77
2022-M-15	Clear Water Control	29.13	83.46	27.60	6.80	4.96
2023-M-13	Clear Water Control	29.20	83.60	27.80	6.75	4.85
2023-M-14	Clear Water Control	29.25	83.50	27.90	6.79	4.88
2023-M-15	Clear Water Control	29.25	83.48	27.92	6.78	4.87

Table S5. Yield of herbicide application under drip irrigation in 2022 and 2023.

Survey Year	Treatments	Active Ingredient Dosage (g a.i./hm²)	Cotton Production (kg/hm²)
2022	48% Flumioxazin SC	50.4	5000.2
2022	48% Flumioxazin SC	50.4	5300.6
2022	48% Flumioxazin SC	50.4	5336.3
2023	48% Flumioxazin SC	50.4	5202.0
2023	48% Flumioxazin SC	50.4	5233.1
2023	48% Flumioxazin SC	50.4	5230.0
2022	48% Flumioxazin SC	100.8	5400.7
2022	48% Flumioxazin SC	100.8	5800.2
2022	48% Flumioxazin SC	100.8	5653.4
2023	48% Flumioxazin SC	100.8	5600.0
2023	48% Flumioxazin SC	100.8	5654.3
2023	48% Flumioxazin SC	100.8	5600.0
2022	42% Fluridone SC	252	5300.7
2022	42% Fluridone SC	252	5500.2
2022	42% Fluridone SC	252	5526.9
2023	42% Fluridone SC	252	5400.0
2023	42% Fluridone SC	252	5527.8
2023	42% Fluridone SC	252	5400.0
2022	50% Prometryn SC	900	5400.7
2022	50% Prometryn SC	900	5700.2
2022	50% Prometryn SC	900	5634.2
2023	50% Prometryn SC	900	5500.0
2023	50% Prometryn SC	900	5600.0

2023	50% Prometryn SC	900	5635.3
2022	Clear water control (CK)	/	4700.4
2022	Clear water control (CK)	/	4950.2
2022	Clear water control (CK)	/	4910.2
2023	Clear water control (CK)	/	4750.0
2023	Clear water control (CK)	/	4950.0
2023	Clear water control (CK)	/	4860.8
